# Altered Mental Status and Quadriparesis in Ambulatory Surgical Care: A Case Report

**DOI:** 10.7759/cureus.86530

**Published:** 2025-06-22

**Authors:** Gonçalo Torrinha, Rui Vieira, Cecília Pacheco, Cecília Vilaça, Sara Bernardo

**Affiliations:** 1 Department of Intensive Care Medicine, Unidade Local de Saúde de Braga, Braga, PRT; 2 School of Medicine, Life and Health Sciences Research Institute (ICVS), University of Minho, Braga, PRT; 3 Department of Anesthesiology, Unidade Local de Saúde de Braga, Braga, PRT

**Keywords:** central venous access, local anesthetic systemic toxicity (last), quadriparesis, secondary pneumothorax, transdermal lidocaine

## Abstract

This case report describes a rare instance of local anesthetic systemic toxicity (LAST) following subcutaneous lidocaine administration for a minor surgical procedure. A 70-year-old male patient developed agitation, dysarthria, and quadriparesis postoperatively, with subsequent investigations revealing a pneumothorax but no major neurological or vascular compromise. The diagnosis of LAST was made after exclusion of other causes. The patient recovered fully within hours without any specific management. This case underscores the need for improved awareness of LAST among non-anesthesiology clinicians and emphasizes preventive strategies for local anesthetic use.

## Introduction

Local anesthetic systemic toxicity (LAST) is a serious and potentially life-threatening complication. This clinical entity is of clear importance in emergency medicine, and presenting symptoms range from seizures, altered mental status, acute respiratory failure, and cardiac arrest [1]. With an estimated two cases per 1,000 nerve blocks, only 32% of overall cases present with isolated central nervous system (CNS) findings [2]. 

Considering its low incidence, it’s important to underline that only 11% of LAST cases arise after subcutaneous infiltration and 11% during intravenous lidocaine administration for analgesia [2]. Nonetheless, lidocaine remains the most commonly implicated agent, probably due to its widespread periprocedural usage [3, 4].

## Case presentation

The Anesthesiology team was activated for a 70-year-old male patient presenting with a sudden onset of agitation and dysarthria. Previously, the patient had been admitted to the ambulatory operating room (OR) for implantation of an intradermal central venous access (Implantofix®, B. Braun Melsungen AG, Melsungen, Germany) for chemotherapy administration, with surgeon-administered local anesthesia and ultrasound and fluoroscopic control. Originally intended to be inserted in the jugular vein, an accidental puncture of the common carotid artery led to needle removal and site compression. A secondary approach was done to the right subclavian vein, where the appearance of an air bubble raised the possibility of accidental lung puncture. The needle was repositioned without removal, and the procedure was carried on. After successful completion of the procedure, while still in the OR, the patient started developing the symptoms that triggered the call for the anesthesiology team. 

Assessment by the team revealed a conscious, oriented, and communicative patient, with a Glasgow Coma Scale score of 15, reporting an overall feeling of “numbness” (sic) and generalized acute-onset weakness. Past medical history included dyslipidemia, benign prostatic hyperplasia, and a recent diagnosis of a gastric tubular adenocarcinoma, Grade 1 pT3N0, with urgent surgical gastrectomy performed one week prior to the event. A former heavy drinker, with reduced consumption since one year prior, the patient’s daily medication consisted of 40 mg pantoprazole intradermal (ID) and 10 mg domperidone three times a day (TID).

Physical examination revealed a sarcopenic patient with palpable subcutaneous emphysema in the upper right anterior quadrant of the thorax, without any evidence of tracheal deviation or hypertensive pneumothorax. Vital signs were taken: heart rate of 60 beats per minute, blood pressure of 110/80 mmHg, body temperature of 36.7ºC, and respiratory rate of 18 cycles per minute with oxygen saturation (SpO2) of 98% without the need for supplementary oxygen. An assessment by neurology was made, describing dysmetria in the finger-to-nose and heel-to-shin tests, flexing cutaneous-plantar reflexes bilaterally, but no language difficulties, cranial nerve findings, nystagmus, major motor deficits, or myoclonus. 

**Figure 1:**
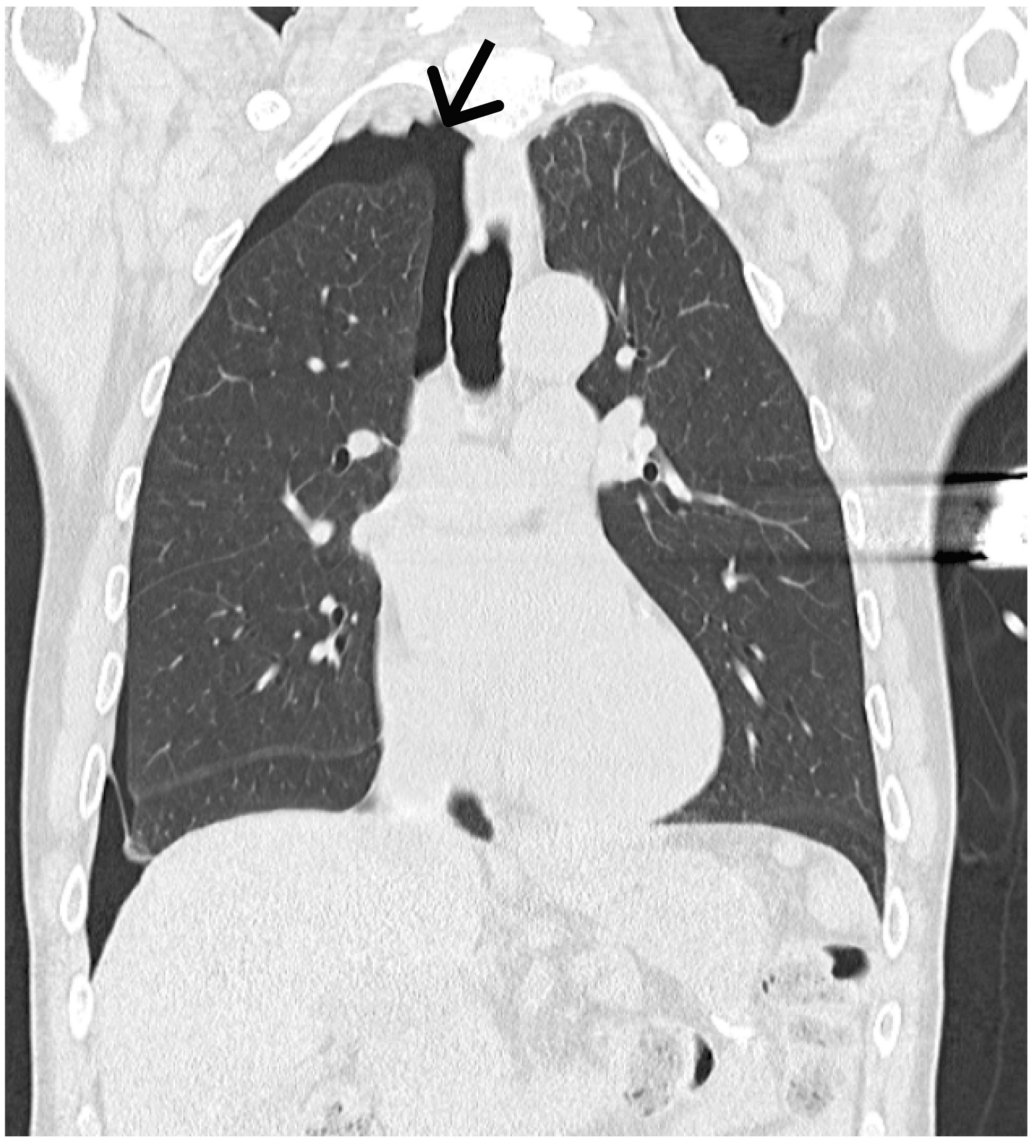
Computerized tomography (CT) scan of the chest evidencing the iatrogenic pneumothorax (arrow) on the patient’s right side in a coronal axis cut.

Head and thorax computed tomography (CT) scans were performed with the complementary administration of contrast. Cranial and cervical studies revealed no acute changes, particularly no occlusion of major vessels. Thoracic CT showed a right pneumothorax with a maximum thickness of 2 cm without any other major findings, namely deviation of structures or vessel occlusion (Figure 1).

Arterial blood gas analysis revealed no significant abnormalities in acid-base balance, ventilation, or oxygenation, with blood ions within normal range and lactate levels under 2 mmol/L. Laboratory tests revealed mild normocytic hypochromic anemia (hemoglobin 10.4 g/dL), mild thrombocytopenia (136,000/µL), a normal leukogram, normal coagulation parameters and kidney function, and negative myocardial infarction serum markers. A 12-lead electrocardiogram showed a sinus rhythm with a frequency of 55 beats per minute, without any evidence of arrhythmias or other conduction abnormalities.

Further inquiry with the surgical team revealed a total administration of 500 mg (25 milliliters) of subcutaneous 2% lidocaine during the procedure and no apparent intravascular plaques or thrombi in the procedural ultrasound.

With the exclusion of other differential diagnoses and considering the total averaged lidocaine infusion of 10 milligrams per kilogram, the team assumed the presumptive diagnosis of LAST, which prompted a consideration for admission to an intermediate care unit. The patient was assessed by the Intensive Care Medicine team, who corroborated all findings, and, after further discussion with the General Surgery team, was placed under oxygen therapy with a high-concentration mask in order to induce pneumothorax absorption in the post-anesthesia recovery unit under close monitoring. Two hours after the event, the patient recovered spontaneously with complete resolution of symptoms and neurological findings, with transfer from the recovery room to the general ward after 10 hours post event and hospital discharge 24 hours later without sequelae or new events. 

## Discussion

With the continuous improvements in surgical care and the change towards ambulatory procedures, it is important to recognize the increasing relevance of the management of perioperative emergencies outside of the ambulatory setting. This presentation of LAST with very specific CNS findings constitutes a rare and atypical manifestation but (directly and indirectly) raises awareness towards such iatrogenic systemic toxicities.

The management of emergencies during ambulatory surgery must take into account not only the usual suspects but also entities specific to anesthesia, including LAST, but also difficult airway, anaphylaxis, and acute ischemic events [5]. In this case, due to the procedure itself, a number of potentially life-threatening differential diagnoses were raised and systematically ruled out, such as hypertensive pneumothorax, pulmonary embolism, stroke, or transient ischemia. This reinforces the current understanding of a need to appropriately plan and train for the management of emergencies, but also calls for an integration of local anesthetics within the potential toxins involved in emergency medicine, namely in peri-arrest and altered mental status algorithms.

Although CNS toxicity is evident in 80% of LAST cases, they aren’t typically isolated [1,2]. Presentation usually includes metallic taste, perioral paresthesias, changes in vision/hearing, dizziness, dysarthria, dysgeusia, agitation, and altered mental status, evolving to seizures and cardiovascular findings in a dose-dependent fashion [1]. This case, therefore, represents a deviation from this norm, especially considering the presence of four-member dysmetria and tetraparesis. A recent systematic review confirms that LAST remains a significant complication in the elderly population and highlights that our understanding of its clinical presentation is still unclear [6]. Out of 19 reports identified in the geriatric population within a period of five years, only three presented after local infiltration, with only one of these presenting with muscle rigidity and altered mental status, as well as cardiac symptoms [7-9]. However, our literature search revealed an additional instance with a more similar presentation [10]. In this case report, a young female patient presented with isolated tetraparesia 30 minutes after the administration of topical lidocaine in the oropharynx in the context of vocal cord cyst surgery. Nonetheless, this case differs from ours in some key aspects. Chen et al. reported a patient with topical application to mucous membranes with coadjuvant administration of epinephrine and dexamethasone, which may have acted to delay the impact of a much lower dosage (200 mg) [10]. In our case, we have to consider that lidocaine could have entered systemic circulation through an intravenous (during the accidental punctures) or subcutaneous route. This could potentially explain the higher speeds of onset and recovery. Additionally, one particular risk factor for LAST, among others, seems to be decreased plasma proteins, validated by our patient’s oncological medical history and evidence of muscle wasting upon physical examination, something that was apparently absent in Chen et al.'s report [10]. Nonetheless, these two cases coincide in their similar clinical presentations (although with different intensities) and exclusion of other potential causes for quadriparesis, namely spinal compression, a complete spinal block, a subdural block, and a spinal cord transient ischemic attack, illustrating the need to consider LAST as a differential diagnosis in such scenarios. 

The case we present also highlights some aspects relevant to appropriate in-hospital management of patients, particularly in the ICU and medical wards. We emphasize the urgent need to address the risks of local anesthetics in perioperative patients, particularly when their management is made by non-anesthesiologists. There is an undeniable increasing trend of minor procedures being performed in medical wards [4, 11]. The lack of systematic education on maximum dosages and the inherent risks of local anesthetics, particularly their various formulations, may lead to an increased incidence of LAST, potentially more common than anticipated. Despite this, LAST remains insufficiently addressed in the differential diagnosis of altered states of consciousness and is absent from advanced life support algorithms and training [12, 13]. This issue is of particular notice in specific medical wards where the involvement of anesthesiologists or intensivists in emergency situations may be infrequent. Therefore, we advocate for the development of internal protocols aimed at preventing local anesthetic overdoses. Such measures should include the preferential use of lower concentration formulations and the incorporation of local anesthetics into the list of toxins to be considered in advanced life support approaches. Additionally, there should be increased awareness of the potential for targeted treatment and reversal using lipid emulsions [1, 14, 15].

## Conclusions

In conclusion, this case of a rare presentation of LAST manifesting as isolated CNS symptoms following a local anesthetic procedure underscores the importance of vigilance for LAST in both traditional and ambulatory surgical settings. Despite the self-resolutory nature of our case, it allows us to underscore the need for improved education on the risks of local anesthetics, particularly among non-surgical specialties, and the need for enhanced awareness and preventive strategies.
